# Epstein-Barr Virus-Negative Post-Transplant Lymphoproliferative Diseases: Three Distinct Cases from a Single Center

**DOI:** 10.4274/Tjh.2012.0010

**Published:** 2014-03-05

**Authors:** Şule Mine Bakanay, Gülşah Kaygusuz, Pervin Topçuoğlu, Şule Şengül, Timur Tunçalı, Kenan Keven, Işınsu Kuzu, Akın Uysal, Mutlu Arat

**Affiliations:** 1 Ankara University School of Medical, Department of Hematology, Ankara, Turkey; 2 Ankara University School of Medical, Department of Pathology, Ankara, Turkey; 3 Ankara University School of Medical, Department of Nephrology, Ankara, Turkey; 4 Ankara University School of Medical, Department of Medical Genetics, Ankara, Turkey

**Keywords:** Renal transplantation, Post-transplant lymphoproliferative disease, Lymphoma, Immunosuppression, Rituximab, Abnormal karyotype

## Abstract

Three cases of Epstein-Barr virus (EBV)-negative post-transplant lymphoproliferative disease that occurred 6 to 8 years after renal transplantation are reported. The patients respectively had gastric mucosa-associated lymphoid tissue lymphoma, gastric diffuse large B-cell lymphoma, and atypical Burkitt lymphoma. Absence of EBV in the tissue samples was demonstrated by both in situ hybridization for EBV early RNA and polymerase chain reaction for EBV DNA. Patients were treated with reduction in immunosuppression and combined chemotherapy plus an anti-CD20 monoclonal antibody, rituximab. Despite the reduction in immunosuppression, patients had stable renal functions without loss of graft functions. The patient with atypical Burkitt lymphoma had an abnormal karyotype, did not respond to treatment completely, and died due to disease progression. The other patients are still alive and in remission 5 and 3 years after diagnosis, respectively. EBV-negative post-transplant lymphoproliferative diseases are usually late-onset and are reported to have poor prognosis. Thus, reduction in immunosuppression is usually not sufficient for treatment and more aggressive approaches like rituximab with combined chemotherapy are required.

## Abstract (tr)

Böbrek naklinden 6-8 yıl sonra Epstein-Barr virus (EBV) negatif posttransplant lenfoproliferatif hastalık geliştiren 3 olgu rapor edilmektedir. Hastaların tanıları gastrik MALT lenfoma, gastrik diffüz büyük B hücreli lenfoma ve atipik-burkitt lenfoma idi. Hem EBV early RNA için yapılan in-situ hibridizasyon yöntemi hem de EBV DNA için yapılan polimeraz zincir reaksiyonu ile doku örneklerinde EBV saptanamamıştır. Hastalara immünsupresyonun azaltılması ile birlikte rituksimab içeren kombine kemoterapi protokolleri verilmiştir. İmmünsupresyonun azaltılmasına rağmen böbrek fonksiyonlarında önemli bir kayıp gözlenmemiştir. Anormal karyotipe sahip atipik Burkitt lenfomalı hasta tedaviye rağmen hastalık ilerlemesi ile erken dönemde kaybedilmiştir. Diğer hastalar hala remisyonda takip edilmektedir. EBV negatif posttransplant lenfoproliferatif hastalıkların genellikle geç başlangıçlı ve kötü prognozlu olduğu bilinmektedir. Bu hastalarda immünsupresyonun azaltılması tek başına yeterli bir tedavi olmadığı gibi rituksimab içeren kombine kemoterapilerle daha agresif tedaviler gerekli olmaktadır.

## INTRODUCTION

Post-transplant lymphoproliferative diseases (PTLDs) are a heterogeneous group of diseases that develop as early or late-onset disease in solid organ or bone marrow transplant recipients with an incidence ranging between 1% and 20% [1,2]. It is reported that in renal transplant recipients PTLD is the second most common malignancy after skin cancer [3,4,5]. Pathogenesis of PTLD is not well understood. Epstein-Barr virus (EBV) infection and prolonged immunosuppression (IS) are the 2 major factors in pathogenesis. Inhibition of cytotoxic T-cell functions due to IS removes the control over EBV-infected B cells, which results in expansion of B cells, acquisition of genetic mutations, and clonal proliferation [6,7]. The highest risk of developing PTLD is within the first year after transplantation. EBV is found to be positive in 60%-80% of PTLDs and is usually associated with early-onset PTLD. However, EBV-negative PTLD is mainly late-onset [8,9,10,11,12]. 

Three patients who had received renal transplantation at other centers were admitted to our department, where they received the diagnosis of PTLD and were further followed. The presence of EBV could not be demonstrated in any of the samples by in situ hybridization for EBV early RNA or polymerase chain reaction (PCR) analysis of EBV DNA. Informed consent was obtained.

## CASE REPORT

**Case 1**

**Figure 1:**
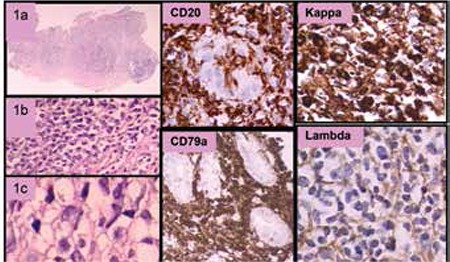
MALT lymphoma. Small monocytoid lymphocytes and some plasma cells in the lamina propria of the gastric mucosa (1a, 1b, 1c). These cells were diffusely positive for CD20 and CD79a, forming a lymphoepithelial lesion. Kappa light chain restriction was demonstrated on neoplastic cells. Bcl10 was negative by immunohistochemistry.

A 28-year-old female patient was diagnosed with Helicobacter pylori (HP)-positive gastric mucosa-associated lymphoid tissue (MALT) lymphoma (stage IE) 6 years after receiving renal transplantation from her mother. She had been on mycophenolate mofetil, tacrolimus, and prednisolone for the last 3 years. After the diagnosis of lymphoma, the immunosuppressive therapy was modified with cessation of mycophenolate mofetil and dose reduction of tacrolimus. The patient received HP eradication therapy and was followed with endoscopic biopsies every 3 months. The HP infection was persistent and all biopsies supported the presence of a MALT lymphoma histologically. One year after the diagnosis, IgH chain clonality analysis by nested PCR revealed 2 separate clones on the polyclonal background, which was consistent with oligoclonal proliferation. The follow-up endoscopy revealed a larger ulcer and the pathology revealed MALT lymphoma invading the muscularis mucosa (Figure 1). The patient responded to combined chemotherapy with rituximab, cyclophosphamide, vincristine, and methyl prednisolone (R-CVP). Five years after diagnosis, she is being followed in complete remission. She is on prednisolone at 5 mg/day per os and tacrolimus at 2 mg/day per os, and her renal functions are stable with serum creatinine levels in the range of 1.3-1.6 mg/dL. 

**Case 2**

**Figure 2:**
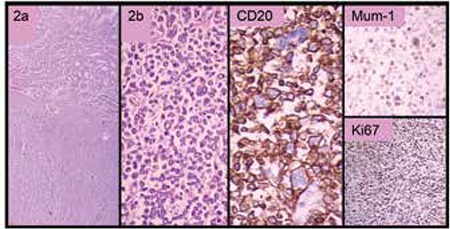
Diffuse large B cell lymphoma diffusely infiltrating the stomach wall. There were also cells that had anaplastic features (2a, 2b). The tumor cells were positive for CD20, were partially positive for MUM-1 and Bcl-6, and had a high proliferation index of around 60% with Ki-67.

A 31-year-old male patient presented with anemia due to chronic blood loss from the gastrointestinal tract. Endoscopic examination revealed a large ulcerated mass of the stomach. A computerized tomography (CT) scan demonstrated an 8.5x4 cm mass extending outside the stomach wall, suggesting an aggressive lymphoma, but endoscopic biopsy revealed MALT lymphoma with lambda monoclonal atypical B cell infiltration. The patient had received renal transplantation 7 years before diagnosis and had been on immunosuppressive therapy with cyclosporine A, azathioprine, and prednisolone since then. After the diagnosis of stage IE PTLD, IS drugs were tapered and stopped. Anti-HP treatment and 6 cycles of combined chemotherapy with R-CVP were administered. Prednisolone at 5 mg/day was resumed due to the deterioration of his renal functions. A CT scan of the abdomen revealed thickening of the stomach wall and perigastric lymphadenopathy. The endoscopy demonstrated the persistence of the giant ulcer in the stomach, with the biopsy revealing diffuse large B-cell lymphoma (DLBCL) (Figure 2). He was treated with total gastrectomy followed by combined chemotherapy with rituximab, cyclophosphamide, vincristine, doxorubicin, and methyl prednisolone, and he is in complete remission 3 years after diagnosis. His serum creatinine levels are in the range of 1.5-2.0 mg/dL with prednisolone at 5 mg/day every other day. 

**Case 3**

**Figure 3:**
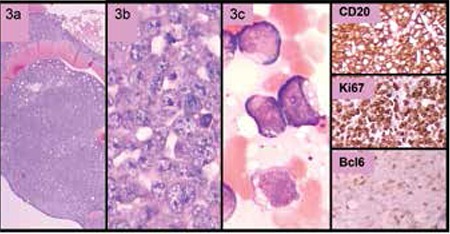
Atypical Burkitt lymphoma. Atypical cells infiltrating the bone marrow had large cytoplasmic vacuoles consistent with L3 morphology (3a, 3b, 3c). The cells were positively stained with CD20, CD79a, and Bcl-6, and were negative for MUM-1 and Bcl-2. The proliferation index examined by Ki-67 was around 100%.

A 28-year-old male patient was admitted to the hospital with ptosis of the left eyelid, diplopia, and extreme sweating for the last few weeks. He had received a renal allograft from a living donor 8 years before. The immunosuppressive regimen consisted of azathiopurine, cyclosporine A, and prednisolone. His complete blood count revealed a white blood cell count of 11.3x109/L, hemoglobin of 15.3 g/dL, and platelet count of 49x109/L. On the peripheral blood smear, 60% of the leukocytes were atypical lymphoid cells with cytoplasmic vacuoles. Bone marrow examination revealed the diagnosis of atypical Burkitt lymphoma (aBL) (Figure 3). Flow cytometric analysis revealed that the immature cells were positive for HLA-DR and the B cell antigens CD19, CD10, cytoplasmic CD22 (+/-), surface CD22, FMC7, CD52, cytoplasmic CD79a, and surface IgM (+/-), and were negative for T-cell antigen CD5. The patient had a complex karyotype consisting of clonal trisomy X, add1 (q25), t(3;6) (q23;q23), trisomy 7, t(8;14) (q22;q32), der (10), der (11), der (16), trisomy 18, trisomy 20, and monosomy 22. Fluorescence in situ hybridization analysis revealed positive results for t(8;14), trisomy 7, and MLL gene amplification. Cerebrospinal fluid examination was negative for malignanT-cells but the magnetic resonance imaging of the brain showed disease infiltration at multiple sites. The patient was treated with discontinuation of the immunosuppressive drugs and with combined chemotherapy that contained rituximab, cyclophosphamide, vincristine, dexamethasone, and L-asparaginase. Central nervous system disease was treated with 6 courses of intrathecal chemotherapy followed by cranial irradiation. His renal functions remained within normal ranges. There was a dramatic clinical response after cessation of IS and initiation of the chemotherapy, and the follow-up bone marrow biopsy after chemotherapy did not reveal any atypical cells. However, several weeks after discharge, he died of disease progression in the central nervous system and medullary relapse.

## DISCUSSION

Three patients with PTLD demonstrating distinct pathologies as well as distinct clinical properties are reported. In all patients, the PTLD occurred long after renal transplantation, and all were EBV-negative. Post-transplant lymphoproliferative diseases may occur as early-onset (≤1 year) or late-onset (>1 year) disease after transplantation [9]. Studies comparing EBV-positive and EBV-negative PTLD patients have demonstrated that EBV-negative PTLD occurred later than EBV-positive PTLD [9,10,14,15,16]. The rarity and the heterogeneity of the disease have prevented large randomized studies to compare the overall survival and treatment outcomes. While some of the studies have shown significantly decreased survival of EBV-negative patients, others could not demonstrate a survival difference [3,9,10,15]. Leblond et al. reported that median survival of EBV-negative patients was significantly shorter than that of EBV-positive patients (1 month vs. 37 months) and identified EBV negativity as an adverse prognostic indicator [9]. On the other hand, Tsai et al. could not demonstrate any significant difference between EBV-positive and EBV-negative PTLD patients both in terms of response to reduction in IS and estimated 1-year overall survival (68% vs. 60% for EBV-positive and EBV-negative groups, respectively) [3]. In accordance with these reports, our cases also had different outcomes: patients 1 and 2 are still alive without disease, while patient 3 had incomplete response to therapy and poor survival. 

Post-transplant lymphoproliferative diseases are very heterogeneous, ranging from early lesions like infectious mononucleosis-like disease to monoclonal monomorphic diseases like malignant lymphomas. The most common type of monomorphic PTLD is DLBCL [14,15]. On the other hand, MALT lymphoma and atypical or Burkitt-like lymphoma are very rarely observed as PTLD [12,13,14]. Immunohistochemical studies demonstrate that PTLD lesions presenting as DLBCL usually express a late germinal center (CD10+/-/bcl-6+/MUM-1-/CD138-) or early post-germinal center (CD10-/bcl-6+\-/MUM-1+/CD138+) profile, but the germinal center profile is more commonly expressed in EBV-negative cases. This may suggest that EBV-negative PTLDs actually resemble the lymphomas that develop in immunocompetent hosts [7,17]. 

 Burkitt lymphoma (BL) or aBL patients typically present with advanced-stage disease and high tumor burden with generalized lymphadenopathy and frequent bone marrow involvement. Typical BL usually has c-myc rearrangement as a sole abnormality. Complex chromosomal abnormalities in addition to c-myc are reported in patients with aggressive B-cell lymphoma with features intermediate between DLBCL and BL, which is also referred to as aBL. In accordance with the literature, patient 3 had very aggressive disease and did not respond to the therapy well [18,19,20,21].

Helicobacter pylori can be demonstrated in the gastric mucosa in a majority of MALT lymphomas, and HP eradication with antibiotics usually results in complete regression. It is not clear whether the risk of MALT lymphoma is increased due to immunosuppression after solid organ transplantation or if it is completely due to HP infection, or both. Similar to case 1, the reported cases in the literature were all negative for EBV and positive for HP, and most developed as late-onset PTLD. They were clinically and histologically identical to conventional MALT lymphomas, which occur in immunocompetent patients [22,23]. Although most post-transplant MALT lymphomas are clinically indolent and do not require aggressive treatment, it is not known whether anti-HP therapy alone is sufficient to treat the post-transplant gastric lymphomas. Nelson et al., in their series of PTLD, reported that a single gastric PTLD that could represent a high-grade MALT lymphoma responded to only reduction in IS [11]. However, our patient did not sufficiently respond to reduction in IS and anti-HP therapy and eventually required combined chemotherapy.

Reduction in IS should be the first line of approach in treatment of PTLD [3]. In the case of kidney transplantation, it is recommended that immunosuppressive therapy should be reduced to a minimum dosage and even ceased as long as the graft rejection is compatible with life. Early lesions and polymorphic PTLD have favorable response to reduction in IS alone, but the monoclonal/monomorphic forms require more aggressive treatment. During the last decade, anti-CD20 monoclonal antibody, rituximab, has become increasingly used in the treatment of CD20+ PTLD with better response rates of up to 60%-70%. Patients who do not respond to reduction in IS and rituximab can be given combined chemotherapy. However, combined chemotherapy with rituximab should be considered as first-line therapy for patients who are not suitable for reduction in IS or who have EBV-negative, late-onset aggressive disease, or for patients who have high tumor burden requiring an upfront rapid intervention [24,25,26,27,28]. In conclusion, EBV-negative PTLDs can be considered as a distinct group of PTLD resembling lymphomas of immunocompetent subjects due to late occurrence, higher proportion of monomorphic cases, and clinically more aggressive behavior.

## CONFLICT OF INTEREST STATEMENT

The authors of this paper have no conflicts of interest, including specific financial interests, relationships, and/ or affiliations relevant to the subject matter or materials included.
